# Effect of Al Concentration on Structural, Optical and Electrical Properties of (Gd, Al) Co-Doped ZnO and Its n-ZnO/p-Si (1 0 0) Heterojunction Structures Prepared via Co-Sputtering Method

**DOI:** 10.3390/ma16062392

**Published:** 2023-03-16

**Authors:** Nur Amaliyana Raship, Siti Nooraya Mohd Tawil, Nafarizal Nayan, Khadijah Ismail

**Affiliations:** 1 Department of Electrical and Electronic Engineering, Universiti Pertahanan Nasional Malaysia (UPNM), Sungai Besi 57000, Kuala Lumpur, Malaysia; 2 Center for Tropicalisation, Universiti Pertahanan Nasional Malaysia (UPNM), Sungai Besi 57000, Kuala Lumpur, Malaysia; 3 Microelectronic and Nanotechnology-Shamsuddin Research Centre (MiNT-SRC), Universiti Tun Hussein Onn Malaysia (UTHM), Parit Raja 86400, Johor, Malaysia

**Keywords:** zinc oxide, rare-earth, Gd and Al co-doping, co-sputtering, heterojunction

## Abstract

Heterojunction structures of n-ZnO/p-Si were prepared through the growth of undoped ZnO and (Gd, Al) co-doped ZnO films onto p-type Si (1 0 0) substrates, using a co-sputtering method. The structural and optical properties of the Gd-doped ZnO films were studied as a function of different Al doping concentrations. The X-ray diffraction profiles indicated that the films had a nanocrystalline structure of ZnO with a (0 0 2) preferential orientation. An increase in the Al doping concentration deteriorated the (0 0 2) diffraction peak intensity. The transmittance measurements in the UV–Vis wavelength range indicated that the film’s optical gap increased with increase in Al doping concentration. The heterojunction parameters were evaluated using the current–voltage (*I-V*) characterization carried out of the fabricated n-ZnO/p-Si heterostructure, in dark conditions at room temperature. From these measurements, the n-ZnO-based DMS/p-Si heterojunction diode with the use of (Gd, Al) co-doped ZnO film showed the lowest leakage current of 1.28 × 10^−8^ A and an ideality factor *η* of 1.11, close to the ideal diode behavior of η = 1, compared to the n-Gd-doped ZnO/p-Si and n-undoped ZnO/p-Si heterojunction diodes.

## 1. Introduction

In-depth research has been performed on the II-VI group semiconductor zinc oxide (ZnO) for a variety of optoelectronic applications, including transparent conducting electrodes (TCO), solar cells, light emitting diodes (LED), UV photodetectors, and heterojunction diodes. This is due to ZnO’s exceptional features, including its broad band gap of 3.37 eV and high exciton binding energy of 60 meV. In this regard, heterojunction diodes have great significance in the field of semiconductors, since they can be utilized in photodiodes as well as solar cells. In order to achieve a heterojunction in ZnO, n-ZnO has been deposited on p-type semiconductors, such as GaN [1], SiC [2], Si [3], and AlGaN [4]. p-GaN has been the material of choice in the majority of ZnO heterojunction research, since both materials share a comparable bandgap, crystal structure of wurtzite, and small mismatch of lattice constant. Other than p-GaN, p-Si is one of the desirable options, due to its affordability, wide area wafers, and most significantly the possibility of integration with well-established Si electronics. From the standpoint of device applications, improving the performance of heterojunction structures is crucial for achieving greater efficiency. The alteration of the structural, optical, and electrical characteristics of ZnO with a suitable dopant may be a practical and efficient method for enhancing device performance. In recent years, rare-earth elements (REEs), including Gd [5,6], Eu [7], Tb [8], Ce [9], Sm [10], Dy [11], and Nd [12], have been reported as dopants of ZnO, in order to create new types of diluted magnetic semiconductors (DMSs). This is because they can significantly improve the conductivity, luminosity, and magnetic properties, with the long lifetime of excited state and multiple valence electrons. Reports are also available on co-doping using different metals including Ag [13], Ga [14], Al [15], and Si [16], with benefits for enhancing the electrical conductivity and widening the transmittance spectrum of DMS materials. However, a controlled manner of utilizing the aforementioned dopants should be established, because they could cause more carriers to scatter inside the lattice, which would lower the device performance. In this regard, Gd is an interesting dopant for the purpose of tuning the properties of ZnO films, while Al has been thought to be the most suitable co-dopant, due to its similarity to Zn in terms of ionic and covalent radii, as well as its additional electron, which may boost ZnO’s conductivity. We believe that the incorporation of both Gd and Al in ZnO will have significant effects on the structural, optical, and electrical properties thereby improving the diodes properties. To the best of our knowledge, there have been no prior papers that specifically addressed the impact of (Gd, Al) co-doped ZnO films on p-n heterojunction diode properties, when prepared via co-sputtering or other techniques. In this current work, we discuss how co-doping ZnO with Gd and Al affects the structural, optical, and electrical properties of p-n heterojunction diodes prepared by means of a co-sputtering technique at room temperature. The structural, optical, and electrical properties were probed using X-ray diffraction (XRD), ultra-violet visible spectroscopy (UV-Vis), and current–voltage (*I-V*) measurements. Electrical parameters such as the leakage current (*I*_o_), barrier height ($\Phi_{B}$), and ideality factor (η) were also evaluated.

## 2. Materials and Methods

### 2.1. Preparation of Undoped ZnO and (Gd, Al) Co-Doped ZnO Films

**Figure 1:**
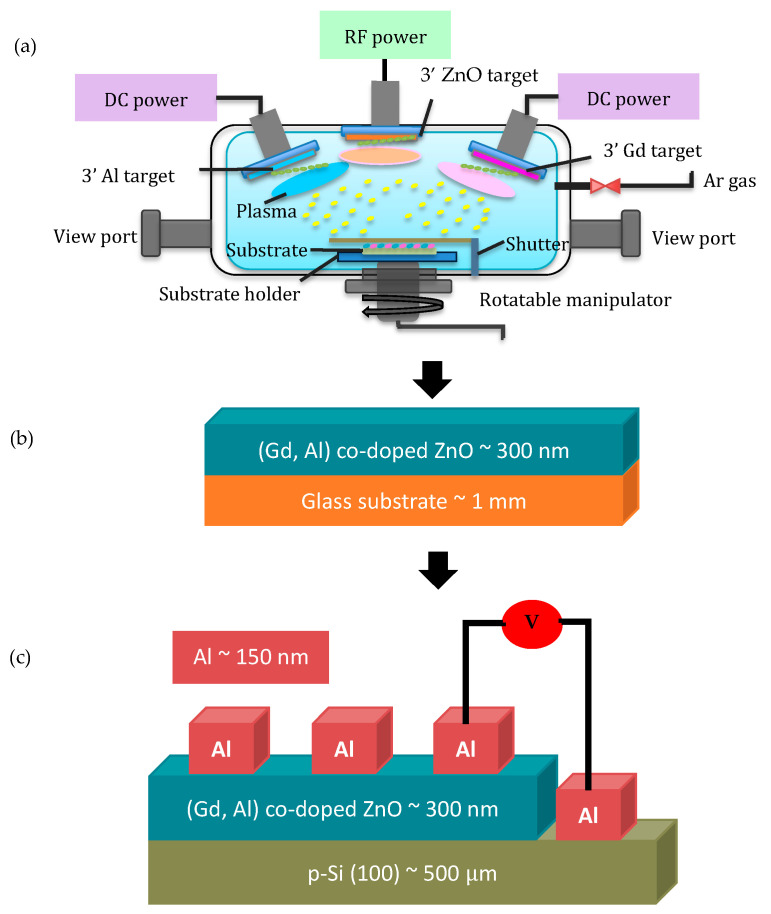
(**a**) Preparation of (Gd, Al) co-doped ZnO films, (**b**) deposited (Gd, Al) co-doped ZnO film, and (**c**) the Al/n- ZnO-based DMS/p-Si heterojunction structure.

The (Gd, Al) co-doped ZnO films with a varied concentration of Al were produced on glass substrates through a co-sputtering approach, using sputtering equipment (SINTEK PSP 5004 (09SN70)). Initially, within the sputtering chamber, a cleaned glass substrate was placed on the plate of the substrate holder. The process of cleaning the substrate was carried out as described in a previous study [17]. The apparatus setup for the co-sputtering process was established with the installation of three targets and with target holders, namely ZnO target (99.99%), Gd target (99.99%), and Al target (99.99%) as illustrated in Figure 1a. Both Gd and Al targets were connected to a DC power supply, while the ZnO target was connected to an RF power supply. The power switch of the machine and the chiller was switched on, to start the sputtering system. The rotary pump was then switched on for 15 min, to warm up the sputtering machine. After that, the rough valve was turned on for about 30 min, to evacuate the chamber at a low vacuum condition. Next, the chamber of the sputtering machine was evacuated under high vacuum conditions by activating the turbo molecular pump (TMP), until the frequency reached 555 Hz. The main valve was then opened and retained a high-pressure condition of a base pressure at 7 × 10^−6^ Torr, to allow the deposition procedure to begin. Argon (Ar) gas was continuously blown into the sputtering chamber at a flow rate of 29 sccm during the deposition process. For 15 min, the target was pre-sputtered, to clean the surface of any contamination, stabilize the system, and achieve the ideal conditions. Other growth conditions of the films were set exactly the same as in our previous report for Gd-doped ZnO [18]. In brief, the power of the ZnO target and working pressure were held constant at 100 W and 10 mTorr, respectively. The distance between the target and the substrate was kept constant at 13.5 cm, and the substrate rotation was fixed at 9 rpm, in order to ensure the homogeneity of the films (Figure 1b) throughout the deposition process. The entire deposition procedure took 60 min at room temperature. The effect of Al doping concentrations from 0 at% to 4 at% were investigated. Different values of dopant concentration were obtained by tuning the power of the Al target from 0 W to 20 W.

### 2.2. Fabrication of Al/n-ZnO-Based DMS/p-Si Structure

Undoped ZnO, Gd-doped ZnO, and (Gd, Al) co-doped ZnO were integrated into n-ZnO-based DMS/p-Si structure, to assess their electrical characteristics with regard to the diode behavior. An illustration of the Al/n-ZnO-based DMS/p-Si (1 0 0) structure is shown in Figure 1c, and the dimension of the film devices was 1 cm^2^. The n-ZnO-based DMS was initially deposited on p-type silicon substrate using a co-sputtering method and was 1 cm × 0.7 cm in size. Electrical contacts with an effective area of 1 cm × 0.1 cm were then made on top of the n-ZnO-based DMS/p-Si structure under vacuum through DC magnetron sputtering of the Al target for 15 min at room temperature. The parameters for the sputtered Al contact are tabulated in Table 1.

### 2.3. Characterization

X-ray diffraction (XRD) with PANalytical X-Pert Powder was used to determine the film’s phases and crystal structure. Scans were performed on all samples by directing Cu Kα radiation at a wavelength of 0.1540 nm at an incidence angle ranging from 20°–80°. The concentrations of Al were determined using energy dispersive X-ray spectroscopy (EDX). Through the use of UV-Vis spectroscopy, both the transmittance and band gap of the films were analyzed. Evaluation of the electrical characteristics of the films was performed using Hall effect measurement (Lakeshore Cryotronics 8400 series). Characterization of the Al/n-ZnO-based DMS/p-Si heterojunction structure was carried out using the *I-V* measurement using a 2-point probe analyzer. The *I-V* measurements were made in a dark at room temperature. The forward bias voltage and reverse bias voltage of Al/n-ZnO-based DMS/p-Si structure were measured from −2.0 V to +2.0 V. By mounting the device on a probe station, measurements of *I-V* were made using Keithley 2400 sources. The data were then extracted from Oriel *I-V* station software and calculated based on the standard *I-V* relation of the diode.

## 3. Results and Discussion

### 3.1. Analysis of XRD

**Figure 2:**
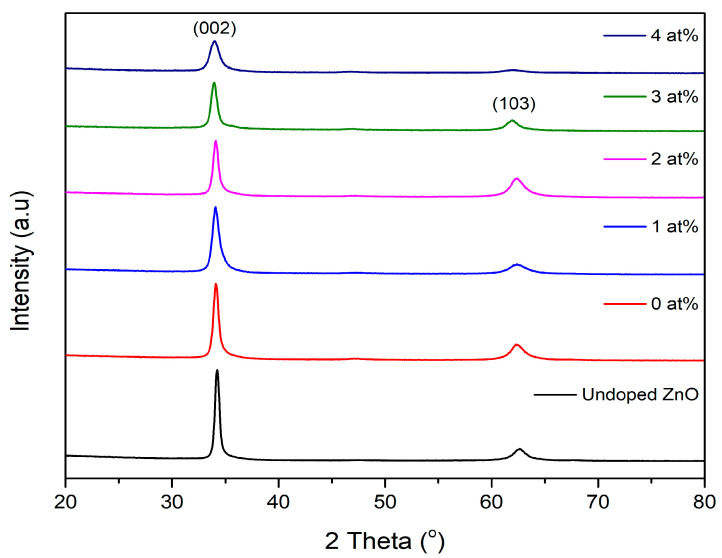
XRD patterns of (Gd, Al) co-doped ZnO films with varied Al concentrations.

The X-ray diffraction spectra of the undoped ZnO and (Gd, Al) co-doped ZnO films with different Al concentrations are depicted in Figure 2. Despite varying the Al concentration, all (Gd, Al) co-doped ZnO films exhibited a ZnO phase with wurtzite structure. Two peaks at (002) and (103) were observed, corresponding to the 2 Theta angles of ~34.0° and ~62.0°. These diffraction peaks were synchronized with the database from the Highscore software’s ICSD file no (98-018-6243). The XRD profiles showed that none of the films had any secondary phases. This meant that the Gd and Al ions successfully replaced Zn ions in the ZnO lattice, to create a single phase structure [19]. When compared to Gd-doped ZnO (0 at% Al), the (002) diffraction peaks of (Gd, Al) co-doped ZnO showed a minor shift in favor of a higher angle as the Al concentration increased. This arose as a result of micro stress within the structure of ZnO and led to the shrinking of the unit cell of ZnO lattice. This can be explained by the smaller Al ionic radii (0.054 nm) replacing the larger Zn ions (0.074 nm), which caused this shifting behavior [20]. Yusoff et al. reported that larger ions shifted the XRD diffraction peak to a lower angle, whereas smaller ions shifted it to a higher angle [21]. This peak shifting was also evidence that the Gd and Al ions had been effectively integrated into the site of the ZnO host.

Using the following equation, the lattice parameters *a* and *c* of the hexagonal wurtzite structure of the films corresponding to the dominant peak (002) were computed.

(1) $$\frac{1}{d_{hkl}{}^{2}}=\frac{4}{3}\;\left(\frac{h^{2}+k^{2}+hk}{a^{2}}\right)+\frac{l^{2}}{c^{2}}$$

where $d_{hkl}$ signifies the interplanar spacing, *a* and *c* denote the constant of lattice parameters of the hexagonal wurtzite structure, and *hkl* represents Miller indices. In Table 2, the computed lattice parameters are provided. The observed *a* and *c* values decreased with the increase of Al concentration. This slight reduction in the values of the lattice parameters can be rationalized by considering the fact that the ionic radius of Al is smaller than ZnO [22]. As observed in the XRD spectra, the dominant (002) peak intensity reduced as the Al concentration of the films was increased. These outcomes are in line with the findings of the study performed by Qian et al. [23]. This suggests that the crystallinity of the films degraded as doping concentration increased. The crystallinity of the films could be confirmed through the resulting value of the degree of crystallinity, which showed that the percentage values decreased when increasing the Al concentration. The XRD spectrum peaks were integrated to assess the degree of crystallinity, by calculating the ratio of the areas under the crystalline (*A_c_*) to the total area of all diffraction patterns, which in this work ranged from a 2 Theta (θ^o^) of 20° to 80° that included the area of crystalline peak (*A_c_*) and the amorphous peak (*A_a_*) [24]. The calculation was based on Equation (2):

(2) $$X_{c}=\frac{A_{c}}{A_{c}+A_{a}}\times 100\%$$

where *A_c_* is the area under the crystalline peak, and *A_a_* is the region with amorphous peaks. Al atoms compete with Zn atoms to obtain additional oxygen atoms and to form more Al-O bonding states, which may slow the crystal development and influence the favored crystallographic plane orientation [23]. The resulting peak intensity was also correlated with the FWHM value, as the FWHM value was even larger at higher Al concentrations. It should be noted that a smaller FWHM value implies that the film had a good crystal quality. The details of the structural parameters of (Gd, Al) co-doped ZnO using different Al concentrations are tabulated in Table 2. Using the dominating peak of (002) as a comparison for all (Gd, Al) co-doped ZnO films, the crystallite size, D, was computed using the Debye-Scherer shown in Equation (3).

D_(002)_ = (0.9 λ)/(β cos θ) (3)

where D denotes the crystallite size, λ is the wavelength of the incident X-ray (0.154 nm), and β represents the observed peak’s full width at half maximum (FWHM). Table 2 shows that the crystallite size ranged between 17 nm and 36 nm, but it decreased as Al concentration increased. This is because dopant atoms disrupt the host ZnO lattice, which slows down the rate of the ZnO crystal formation and nucleation [19]. From the resulting crystallite size, the dislocation density (δ) could be determined through Smallman’s Equation (4).

δ = 1/D^2^ (4)

The dislocation density increased with the increase in Al concentration, which shows that there were more defects in the lattice with higher Al concentrations. Almoussawi et al. reported that degradation of crystallinity at a higher amount of co-doping leads to an increase in dislocation density [25]. Thus, the decrease of film crystallinity with higher co-doping concentrations is associated with the increase in dislocation density. The Stoke Wilson equation was used to determine the amount of microstrain in the film that could be attributed to the lattice defects and distortion [26]. The Stoke Wilson equation is indicated as follows:

(5) $$\varepsilon =\frac{\beta }{4\tan\theta }$$

The undoped ZnO film had a microstrain value of 2.95 × 10^−3^. As tabulated in Table 2, the microstrain value showed an increase with the increase of Al doping concentration. The ionic radius of Gd and Al ions was related to the change that caused the microstrain in the doped films, whereby a lattice disorder was produced when the Zn ions were replaced by Gd and Al ions, which eventually increased the microstrain of the lattice [27,28].

### 3.2. EDX Analysis

**Figure 3:**
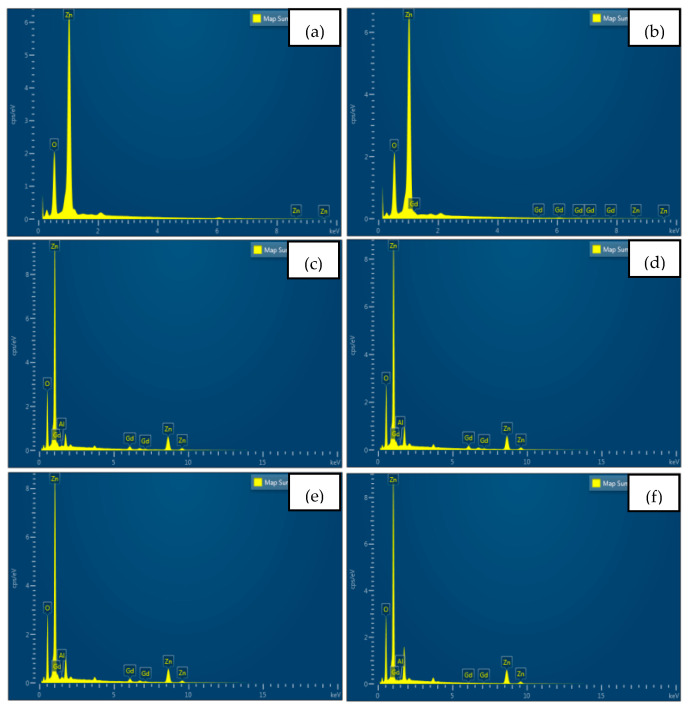
EDX spectrum of (**a**) Undoped ZnO, and (Gd, Al) co-doped ZnO at (**b**) 0 at% Al, (**c**) 1 at% Al, (**d**) 2 at% Al, (**e**) 3 at% Al, (**f**) 4 at% Al.

Figure 3 displays the EDX spectrum of the chemical composition of (a) undoped ZnO and (Gd, Al) co-doped ZnO films at (b) 0 at% Al, (c) 1 at% Al, (d) 2 at% Al, (e) 3 at% Al, and (f) 4 at% Al. Figure 3a,b show the EDX spectrum, which indicates that undoped ZnO contained Zn and O elements, while the film at 0 at% Al contained Zn, O, and Gd elements. For 1 at% Al to 4 at% Al, the EDX spectrum revealed the existence of Zn, O, Gd, and Al, which are confirmed by the EDX spectrum in Figure 3c–f. The results also show that the films did not contain other elements, demonstrating the successful doping of Gd and Al ions into the host Zn sites. This finding matches the result from XRD, since no secondary peaks were detected. The element composition is tabulated in Table 3.

### 3.3. UV-Vis Analysis

**Figure 4:**
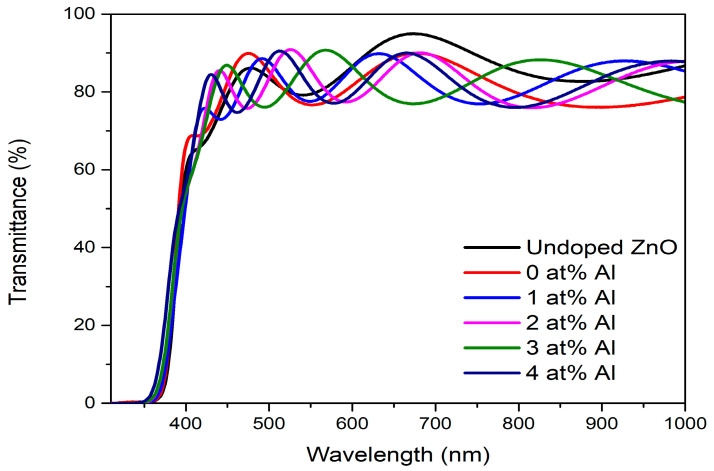
Transmittance spectrum of (Gd, Al) co-doped ZnO films with the variation of the Al concentration.

Figure 4 depicts the transmission spectra of undoped ZnO and (Gd, Al) co-doped ZnO films with varying amounts of Al. The findings demonstrated that all films had good transparency, which transmitting over 80% on average in the visible region. The average transmittances were about 83.8%, 82.3%, 82.1%, 82.0%, 81.7%, and 80.8% for undoped ZnO, with 0 at%, 1 at%, 2 at%, 3 at%, and 4 at%, respectively. The values were not much different but slightly decreased as the Al concentrations rose. The reduction in transmittance was caused by the addition of more dopant ions, due to the structural disorder, where the XRD peak was distorted as a result of increased scattering of light at higher doping. This data thus supports the inference of the structural properties from XRD, which demonstrates that a good transmittance was achieved in the films characterized by high crystallinity. Moreover, the transmittance spectra demonstrated that as the Al doping concentrations were increased, the sharp absorption edges in the 350–385 nm region slightly shifted toward a shorter wavelength. Consequently, the band gap enlarged. This blue shifting behavior of the films may have been caused by the interaction of *sp-f* and *sp-d* among the band electrons and by the localized *f* and *d* electrons of the Gd and Al ions replacing the Zn ions [29].

**Figure 5:**
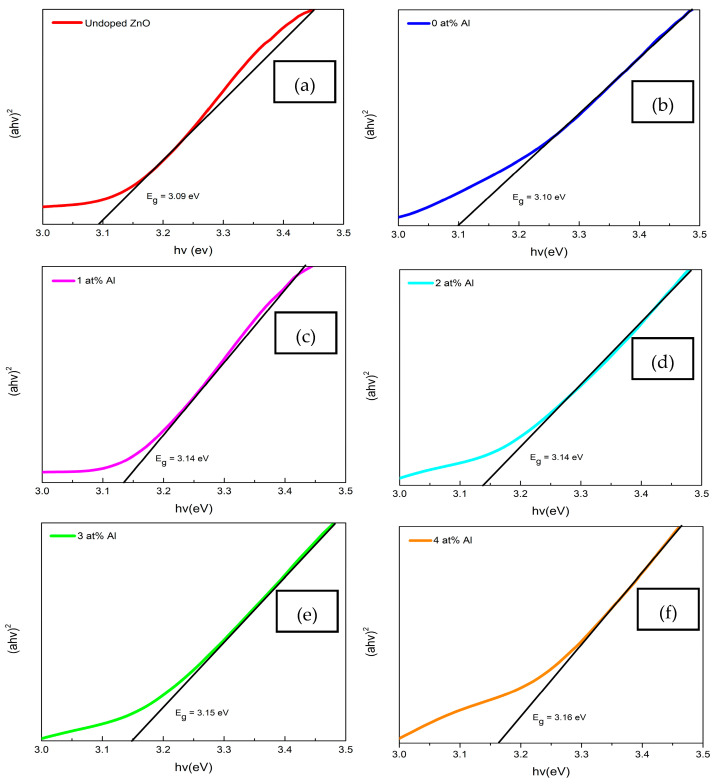
Extrapolation linear plot of band gap energy from Tauc’s plot equation of (**a**) Undoped ZnO, and (Gd, Al) co-doped ZnO films at different Al concentrations of (**b**) 0 at% Al, (**c**) 1 at% Al, (**d**) 2 at% Al, (**e**) 3 at% Al, and (**f**) 4 at% Al.

**Figure 6:**
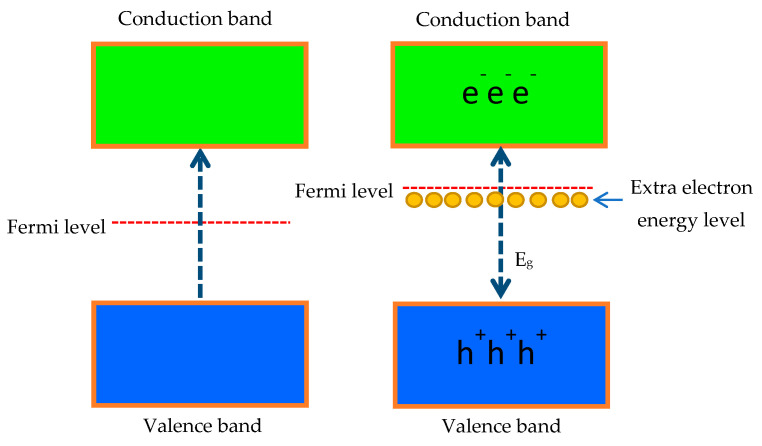
Schematic diagram of transition electron energy level from the valence to conduction band.

Figure 5 demonstrates the plot of (α*hν*)^2^ versus photon energy (*hv*) for (Gd, Al) co-doped ZnO. The band gap was computed by projecting the linear section of (α*hν*)^2^ to zero using Tauc’s plot model, as shown in Equation (6).

(6) $$\alpha hv=A{\left(hv-E_{g}\right)}^{n}$$

where α denotes the absorption coefficient, *A* is a constant value, *E_g_* represents the band-gap energy, *hv* stands for the photon energy, and n = 0.5 corresponds to the direct band-gap, while the indirect band-gap is n = 2. As ZnO is typically categorized as a direct band-gap material, n = 0.5 was chosen as the constant for these computations [30]. The calculated energy band gap values of the 0 at%, 1 at%, 2 at%, 3 at%, and 4 at% of (Gd, Al) co-doped ZnO films were 3.10 eV, 3.14 eV, 3.14 eV, 3.15 eV, and 3.16 eV, respectively. It was discovered that the band gap of co-doped ZnO films slightly increased over that of the 0 at% Al. This observed red shift with Gd and Al ions in the ZnO matrix is commonly related to the interaction of *sp-d* connecting the band electrons and the localized *d*-electrons of the Gd and Al ions that replace the Zn ions. Particle size reduction induced the expansion of the band gap, which occurred due to the quantum confinement phenomenon, as reported by Obeid et al. [31]. This result was comparable with the analysis using XRD and FESEM, which showed that the crystallite size decreased with Gd and Al doping. Moreover, the band gap expansion can be explained by the Burstein-Moss phenomenon. Al and Gd atoms replaced the Zn ions as donors and released electrons that were positioned in the conduction band’s bottom state. The donor Al atoms provided additional carriers, thus causing the Fermi level increases and a shift close to the conduction band. It took more energy for the electrons in the valence band to reach the higher energy state in the conduction band of the (Gd, Al) co-doped ZnO, assuming that the vertical electron transition moved from the valence band to the conduction band as illustrated in Figure 6. As a result, the presence of Al doping moved the conduction band up, which made the band gap value even larger.

### 3.4. Electrical Properties

**Figure 7:**
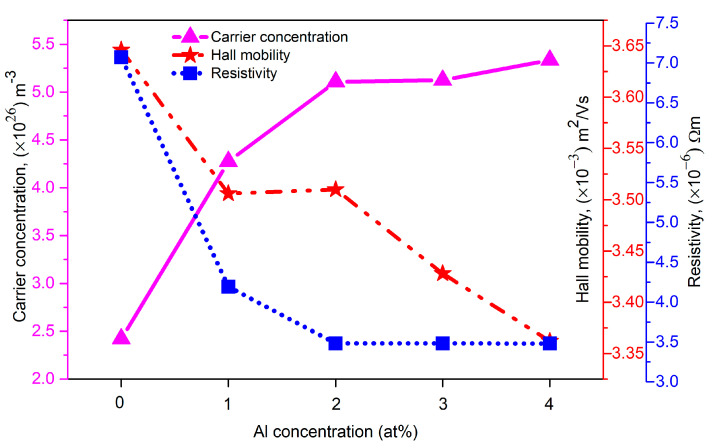
The hall mobility, carrier concentration, and resistivity (Gd, Al) of co-doped ZnO films at various Al concentrations.

Figure 7 presents the electrical characteristics of (Gd, Al) co-doped ZnO films with varying amounts of Al. The following equation was used to represent the relationship that existed among the carrier concentration, hall mobility, and resistivity.

(7) $$\rho =\frac{1}{qn\mu }$$

where ρ represents the resistivity, q denotes the charge of the electron, n is the carrier concentration, and μ stands for the hall mobility. Detailed values of the electrical properties are tabulated in Table 4 and were extracted using Lakeshore Cryotronics 8400 series software. The carrier concentration significantly improved from 2.421 $\times \;10^{26}{\;m}^{-3}$ to 5.334 $\times \;10^{26}{\;m}^{-3}$ as the amount of Al doping was increased from 0 at% to 2 at%, indicating a high doping efficiency at this site. With the incorporation of Al^3+^ into Zn^2+^, these films could generate free electrons. This mechanism is explained by the following equation [32]:

(8) $${\left({\mathrm{Zn}}_{\mathrm{Zn}}\right)}^{X}+\mathrm{Al}\;\to \;\left({\mathrm{Al}}_{\mathrm{Zn}}\right)+\mathrm{Zn}+e^{-}$$

where the Zn at the original lattice sites is denoted by (Zn_Zn_)^X^, while the Al^3+^ in the Zn^2+^ site is denoted by (Al_Zn_). As a result, the carrier concentration increased along with the Al doping concentration. However, a slight increase in the value of the carrier concentration was observed when the Al doping level was increased further, from 2 at% to 4 at%. According to this behavior, when the Al doping level is more than 1 at%, partial Al atoms begin to coalesce from the ZnO lattice. A similar behavior was seen in (Al, Co) co-doped ZnO films [32]. Contrarily, the carrier mobility showed an inverse relationship with the carrier concentration. The lower carrier mobility with increased Al concentrations was mostly caused by the decrease in the crystallinity of the film. For films with a high Al concentration, increased grain boundaries strengthened the carrier scattering in the ZnO. However, the change in carrier mobility was not significantly different from the variation in carrier concentration, i.e., about 3 $\times 10^{-3}\;m^{2}/\mathrm{Vs}$, which shows a slight decrease. The electrical resistivity decreased with increasing Al doping concentrations, as seen in Figure 7. This was caused by doping with trivalent atoms of Gd and Al, which induced large charge carriers, which in turn raised the conductivity of the semiconductor and lowered its resistivity. This could also have been as consequence of the rise in carrier concentrations brought on by the decreased grain boundary scattering in the films [33]. These results are associated with the XRD data, since there was a decrease in crystallite size as the amount of Al was increased, resulting in less scattering at the grain boundary and, thus, a decrease in electrical resistivity [34]. Therefore, the resistivity of the film was primarily influenced by the carrier concentration.

### 3.5. Current-Voltage Characteristics of Al/n-ZnO-Based DMS/p-Si Structure

**Figure 8:**
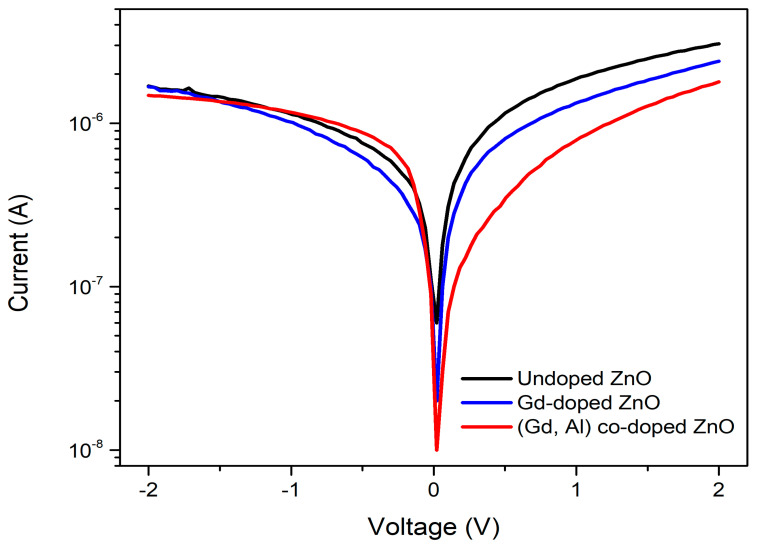
*I-V* measurements of the n-ZnO-based DMS/p-Si heterojunction.

Measurements of current–voltage (*I-V*) were used to ascertain the electrical properties of the heterojunctions. Figure 8 shows a semi-logarithmic plot of the *I-V* measurements of the n-ZnO-based DMS/p-Si heterojunction in the dark. Table 5 provides a summary of the electrical characteristics of n-ZnO-based DMS/p-Si heterojunction derived through *I-V* measurements. The forward and reverse bias *I-V* characteristics of both the forward and reverse bias of the n-ZnO-based DMS/p-Si heterojunctions were assessed using a voltage between +2 V to −2 V at room temperature. In accordance with the p–n junction theory, the following equation describes the typical *I-V* relationship of a diode [35]:

(9) $$I=I_{o\;}\exp\left[\frac{qV}{\eta kT}-1\right]$$

where *V* stands for voltage bias and *I*_o_ denotes saturation current, often known as the leakage current, and which is calculated by taking the straight line intercept of ln *I* at *V* = 0. The equation of saturation current *I*_o_ is given by

(10) $$I_{o}=AA^{*}T^{2}\exp\left(\frac{q\Phi_{B}}{kT}\right)$$

where *A* is the effective contact area, A* is the constant of Richardson, *q* denotes charge, *k* is Boltzmann’s constant, $\Phi_{B}$ denotes barrier height, and *T* stands for temperature. The leakage current of n-undoped ZnO/p-Si, n-Gd-doped ZnO/p-Si, and n-(Gd, Al) co-doped ZnO/p-Si were found to be 4.1 × 10^−7^ A, 1.13 × 10^−6^ A, and 2.0 × 10^−8^ A, respectively. A low leakage current was observed in the (Gd, Al) co-doped ZnO film, which was beneficial to reduce loss as less heat was trapped [36]. Similar values for the leakage current were discovered in the present study and in the work by Bedia et al. [35], where the n-ZnO/p-Si heterojunction was 8.01 × 10^−8^ A.

The following equation was used to compute the barrier height using the saturation current *I*_o_, which was derived by extrapolating the linear portion of the semi-logarithmic forward *I–V* curves to zero applied voltage.

(11) $$\Phi_{B}=\frac{kT}{q}\ln\left(\frac{AA^{*}T^{2}}{I_{0}}\right)$$

The barrier heights for n-undoped ZnO/p-Si, n-Gd-doped ZnO/p-Si, and n-(Gd, Al) co-doped ZnO/p-Si heterojunction structures were 0.71 eV, 0.76 eV, and 0.78 eV, respectively. The barrier heights increased when doping with Gd, as well as (Gd, Al), and the largest barrier height was observed for the n-(Gd, Al) co-doped ZnO/p-Si heterojunction. This indicates that the addition of doping element changed the effective barrier height and modified the p-Si space charge area [5]. A higher barrier height also contributed to the enhancement of the interface quality of the junction. Gd:ZnO/p-Si junctions were made by Baturay et al. [5] by depositing undoped ZnO, 1%, 3%, and 5% Gd-doped ZnO thin films. The barrier height for the ZnO/p-Si junctions and Gd:ZnO/p-Si junctions were both 0.707 eV and ranged between 0.815 eV to 0.836 eV for ZnO films that were 1% to 5% doped with Gd.

The ideality factors of heterojunctions are listed in Table 4 and were calculated using the following equation from the slope of forward bias ln *I-V* plot’s linear area.

(12) $$\eta =\frac{q}{kT}\frac{\mathrm{dV}}{d(\ln I)}$$

The heterojunction of n-undoped ZnO/p-Si had an ideality factor, η = 1.66, while the ideality factors of the n-Gd-doped ZnO/p-Si, and n-(Gd, Al) co-doped ZnO/p-Si were 1.24 and 1.11, respectively. A good diode should have an ideality factor that is close to one [37]. When the ideality factor is more than or equal to two (η ≥ 2), it dominates through the recombination current, while if the ideality factor is equal to one (η = 1), it dominates through diffusion current. The doping element consequently had an impact on the heterostructure’s charge transport mechanism. The fact that there was an ideality factor more than one indicated that the diode was not behaving in the ideal manner. This behavior was shown due to the distribution of interface states, recombination of electrons and holes in the depletion region, and/or due to the increase in the diffusion current by the increasing applied voltage [38]. The reduction in the ideality factor after Gd doping and co-doping with Gd and Al demonstrated that the doping approach enhanced the n-undoped ZnO/p-Si interface and changed the non-ideal diode characteristics to a nearly ideal diode behavior. As reported by Sasikumar et al. [39], smaller crystallites in the films, inhomogeneities, and reduction of non-uniform distribution of the interfacial charges contributed to an improvement of the ideality factor of heterostructures. Thus, their study is comparable with our study, as the co-doping films exhibited a smaller crystallite size, which in turns improved the ideality factor towards one. A similar result trend was reported by Yakuphanoglu et al. [40], who produced heterojunctions of p-Si/n-undoped ZnO and p-Si/n-B-doped ZnO using a spin coating method. The measurement of the ideality factor of ZnO thin films doped with boron was found to be lower, and heterojunctions rectifying characteristics were enhanced by doping with boron.

**Figure 9:**
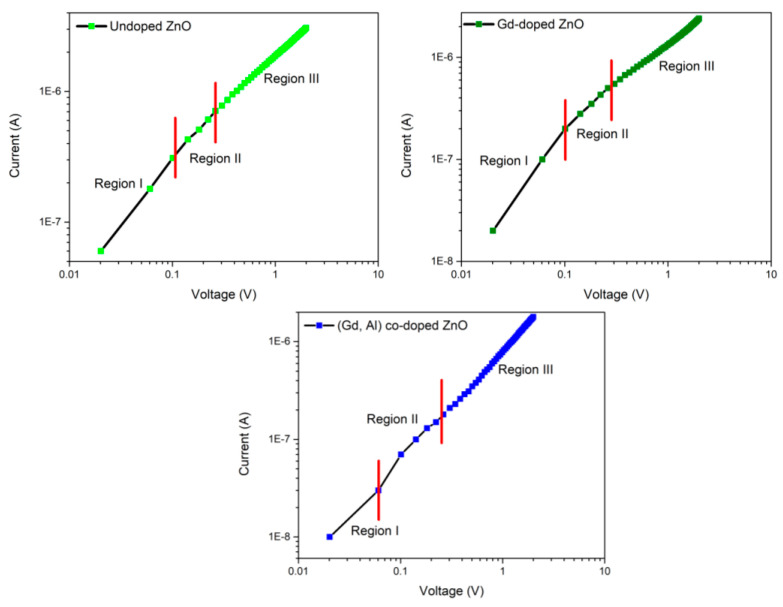
Double logarithmic of *I-V* measurements for the n-ZnO-based DMS/p-Si heterojunction.

The plotting of the logarithmic of current (*I*) versus voltage (*V*), as shown in Figure 9, was to determine the current transport mechanism of the n-ZnO-based DMS/p-Si heterojunction. As shown in the graph plotted in Figure 9, the n-undoped ZnO/p-Si, n-Gd-doped ZnO/p-Si, and n-(Gd, Al) co-doped ZnO/p-Si heterojunction structures had three distinct regions. Region I, with a low forward voltage and less than 0.1 eV, denotes the ohmic behavior in which the current is linearly dependent on the voltage. This ohmic current transport mechanism indicates that the charge carriers were transported via tunneling between the two states at the interface [3]. Region II represents the intermediate forward voltage around 0.1 < *V* < 1. The current observed in this region grew exponentially with voltage according to equation *I*~exp *V*, suggesting that recombination tunneling may be the underlying transport mechanism [10]. This mechanism is typically seen in wide band gap semiconductors. At higher voltages, in region III, the current was dependent on the power law (*I*~*V*^2^), indicating a space-charge-limited current (SCLC) transport mechanism. The SCLC mechanism took place because of the existence of traps in the ZnO band gap [9].

**Figure 10:**
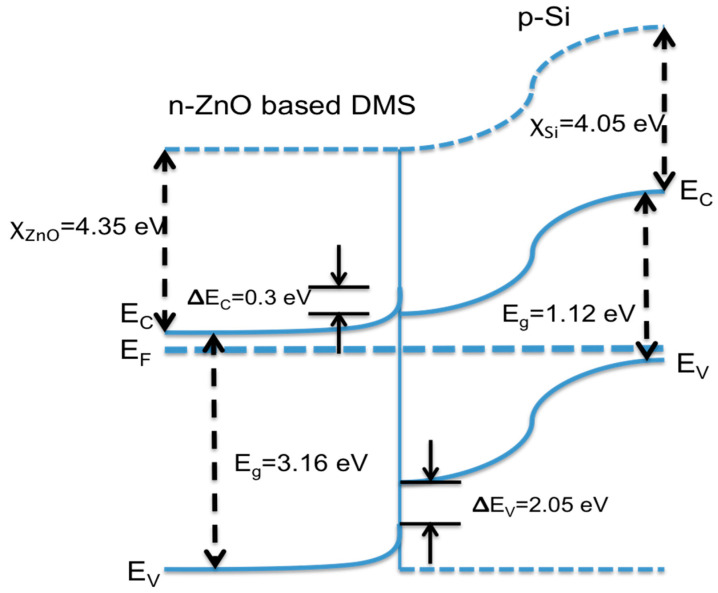
Energy band diagram for the n-ZnO-based DMS/p-Si heterojunction.

Figure 10 depicts the heterojunction n-ZnO-based DMS/p-Si of energy band diagram created using the Anderson model under a state of thermal equilibrium [41]. In order to generate the band diagram, it was presumed that the electron affinities were 4.35 eV and 4.05 eV for ZnO and Si, respectively [42]. The general energy band gap for Si was 1.12 eV, while the energy band gap of the ZnO-based DMS was 3.16 eV, computed by projecting the linear section of (α*hν*)^2^ to zero using Tauc’s plot model, as discussed earlier in the optical properties section. A band offset existed between the conduction and valence bands, which was the result of the differences in the affinities of the electrons and the band gap, where the conduction band offset, $\Delta E_{C}$, and valence band offset, $\Delta E_{V}$, were 0.3 eV and 2.05 eV, respectively. Both band offsets gathered the electrons and holes at the interface. Small values of $\Delta E_{C}$ mean the electrons may move from the Si conduction band to the ZnO conduction band. Nevertheless, a higher value of $\Delta E_{V}$ in the valence band inhibits holes from moving from ZnO to Si. Since the carrier is greater in p-Si than in ZnO, the depletion area will predominantly be expanded in ZnO. As a consequence, the flow of electrons from n-ZnO dominates the current transport in the heterojunction. By applying a forward bias, the potential barrier between ZnO and p-Si is reduced, allowing electrons to flow more freely from ZnO into p-Si. As a result, the forward current increases progressively with the increase of the voltage bias.

## 4. Conclusions

In conclusion, varied Al concentrations of (Gd, Al) co-doped with ZnO were successfully deposited using a co-sputtering method. XRD analysis revealed no secondary phases in any of the films, showing the success of the Gd and Al ion substitutions into the Zn ions. The optical transmittance spectra for all films were above 80%, according to the UV-Vis measurements, and the optical band-gap showed a red-shift with the increasing Al content. The electrical properties showed Al co-doping improved the carrier concentration and reduced the resistivity of the Gd-doped ZnO film. The effect of Al doping on (Gd, Al) the co-doped ZnO film was demonstrated by the performance of the n-ZnO/p-Si heterojunctions diode. The results indicated a low leakage current of 1.02 × 10^−8^ A and an ideality factor, η, of 1.11, approaching the ideal diode behavior of η = 1. Thus, this demonstrated the possibility of using the prepared films for future DMS-based electronic devices.

## Figures and Tables

**Table 1:** List of parameters for the Al electrical contact.

Parameter	Value
Base pressure	5 × 10^−6^ Torr
Argon flow rate	100 sccm
Distance target to substrate	12.0 cm
Rotating substrate holder	10 rpm
Working pressure	5 mTorr
Sputtering power	100 W
Temperature	Room temperature
Deposition time	15 min

**Table 2:** Structural properties of (Gd, Al) co-doped ZnO films with variation of the Al concentration.

Al Concentrations (at %)	Lattice Constant (Å)		Crystallite Size, *D* (nm)	FWHM (2 θ°)	Degree of Crystallinity (%)	Microstrain ε (10^−3^ Lin^−2^ m^−2^)	Dislocation Density δ (10^−4^ nm^−2^)
	*a*	*c*					
Undoped ZnO	3.045	5.275	42.00	0.2066	45.76	2.95	5.67
0	3.043	5.270	36.73	0.2362	43.42	3.37	7.41
1	3.037	5.260	26.78	0.3240	36.95	4.62	13.94
2	3.039	5.264	21.00	0.4133	36.18	5.89	22.69
3	3.039	5.262	18.54	0.4680	33.53	6.67	29.09
4	3.033	5.254	17.30	0.5018	21.09	7.14	33.43

**Table 3:** The composition of (Gd, Al) co-doped ZnO films at various Al concentrations.

Al Concentration (at%)	Gd (at%)	Al (at%)	Zn (at%)	O (at%)
Undoped ZnO	NA	NA	47.95	52.05
0	3.22	NA	43.27	53.51
1	3.09	1.17	41.91	53.83
2	2.91	2.40	40.68	53.91
3	3.40	3.05	39.53	54.02
4	3.16	3.97	38.89	53.98

**Table 4:** The electrical values of (Gd, Al) co-doped ZnO films at various Al concentrations.

Al Concentration (at%)	Carrier Concentration ×10^26^ (1/m^3^)	Hall Mobility ×10^−3^ (m^2^/V·s)	Resistivity ×10^−6^ Ω·m
0	2.421	3.646	7.070
1	4.275	3.506	4.193
2	5.108	3.510	3.482
3	5.124	3.428	3.482
4	5.334	3.362	3.480

**Table 5:** The electrical characteristics of n-ZnO-based DMS/p-Si heterojunction derived from *I-V* measurements.

Sample	Leakage Current, *I*_o_ (A)	Ideality Factor (*η*)	$\mathrm{Barrier}\;\mathrm{Height,}\;\Phi_{B}$ $\left(eV\right)$
Undoped ZnO	6.11 × 10^−8^	1.66	0.74
Gd-doped ZnO (0 at% Al)	2.09 × 10^−8^	1.24	0.76
(Gd, Al) co-doped ZnO (3 at% Al)	1.02 × 10^−8^	1.11	0.78

## Data Availability

Data are contained within the article.
